# Real-World Effectiveness and Safety of Upadacitinib in Tumor Necrosis Factor-Inhibitor Refractory Axial Spondyloarthritis: 52-Week Outcomes from a Single-Center Cohort

**DOI:** 10.5152/ArchRheumatol.2025.11098

**Published:** 2025-12-01

**Authors:** Umut Bakay, Tuğba İzci Duran

**Affiliations:** 1 Department of Rheumatology, Denizli State Hospital, Denizli, Türkiye

**Keywords:** Axial spondyloarthritis, enthesitis, JAK inhibitors, real-world study, upadacitinib

## Abstract

**Background/Aims:**

Patients with axial spondyloarthritis (axSpA) who fail tumor necrosis factor inhibitors (TNFi) represent a treatment-refractory subgroup with limited options. Real-world evidence (RWE) on the effectiveness and safety of selective Janus kinase 1 (JAK1) inhibition in this setting remains scarce. This study aimed to evaluate the 52-week real-world effectiveness, persistence, and safety of upadacitinib in TNFi-refractory axSpA patients.

**Materials and Methods:**

In this retrospective, single-center study, 45 Turkish axSpA patients with prior TNFi failure received upadacitinib 15 mg daily and were assessed at weeks 12, 24, and 52. Effectiveness outcomes included Bath Ankylosing Spondylitis Disease Activity Index (BASDAI), Ankylosing Spondylitis Disease Activity Score (ASDAS), Bath Ankylosing Spondylitis Functional Index (BASFI), Bath Ankylosing Spondylitis Metrology Index (BASMI), Health Assessment Questionnaire (HAQ), Visual Analog Scale for pain, and Assessment of SpondyloArthritis International Society (ASAS) 20/40/70 responses analyzed with non-responder imputation (NRI). Treatment persistence was evaluated by Kaplan–Meier analysis, predictors of discontinuation by Cox regression, and safety outcomes as incidence per 100 patient-years (PY).

**Results:**

Mean age was 48.1 years; 53.3% were male; 68.9% human leukocyte antigen B27 positive. At week 52, BASDAI and ASDAS decreased significantly (both *P* < .001), with parallel improvements in BASFI, BASMI, HAQ, and pain. The ASAS20/40/70 responses were achieved by 75.6%, 60.0%, and 26.7% of patients (NRI). Drug retention was 75.6% at 1 year; discontinuations occurred due to adverse events (AEs) (4.4%), treatment failure (11.1%), patient decision (6.7%), and pregnancy (2.2%). Prior interleukin-17 inhibitor (IL-17i) exposure (HR 3.88) and multiple TNFi use (HR 3.02) predicted shorter drug survival. Across 38.3 PY, no serious infections, malignancies, or thromboembolic events were observed; AEs were infrequent and manageable.

**Conclusion:**

Upadacitinib provided sustained improvements in disease activity, function, and patient-reported outcomes in TNFi-refractory axSpA, with favorable persistence and safety. Despite limitations of the retrospective, single-center design and modest sample size, these findings complement pivotal trials and global real-world data and represent the first RWE of upadacitinib use in Türkiye, supporting JAK1 inhibition as a therapeutic option in difficult-to-treat populations.

**Main Points**

- Upadacitinib achieved rapid and durable improvements across disease activity, functional capacity, and patient-reported outcomes over 52 weeks in tumor necrosis factor inhibitor (TNFi)-refractory axial spondyloarthritis.
- At week 52, Assessment of SpondyloArthritis International Society (ASAS)20, ASAS40, and ASAS70 responses were 75.6%, 60.0%, and 26.7%, respectively, confirming both early and sustained clinical benefit.
- Drug retention at 1 year was 75.6%, with prior interleukin-17 inhibitor exposure and multiple TNFi use predicting higher risk of discontinuation.
- The safety profile was favorable and consistent with pivotal trials, with no serious infections, malignancies, or thromboembolic events observed.
- This study provides the first real-world evidence from Türkiye, where strict reimbursement rules mandate documented TNFi failure before Janus kinase inhibitor initiation, thereby focusing on a uniquely treatment-refractory cohort with high unmet needs.

## Introduction

Axial spondyloarthritis (axSpA) is a chronic, immune-mediated inflammatory disorder that predominantly involves the sacroiliac joints and the spinal column.^1^ In recent years, epidemiological studies have reported that the global prevalence of axSpA ranges from 0.3% to 1.4%, with significant inter-country variability influenced by ethnicity and diagnostic practices.^2^^,^^3^ In Türkiye, the rising number of rheumatologists and increasing awareness of axSpA among non-rheumatology specialties have likely contributed to improved detection and a higher reported prevalence in recent years. A population-based study conducted in İzmir using the Assessment of SpondyloArthritis International Society (ASAS) classification criteria reported an axSpA prevalence of 1.3%.^4^ This figure approaches the upper limit of global estimates and reflects a substantial disease burden in urban regions of Türkiye, underscoring the need for optimized diagnostic and therapeutic strategies.

The disease encompasses both radiographic, ankylosing spondylitis, (AS) and non-radiographic (nr-axSpA) forms, which share key clinical features such as inflammatory back pain, morning stiffness, enthesitis, and extra-articular manifestations including uveitis and psoriasis.^5^ Delays in early diagnosis and treatment may result in irreversible structural damage, persistent pain, and significant impairments in physical function and quality of life.^6^^,^^7^ Although nonsteroidal anti-inflammatory drugs (NSAIDs) are the standard initial therapy for axSpA, contemporary treatment recommendations—from bodies such as ACR, EULAR, ASAS/EULAR, the British Society for Rheumatology, and regional guidelines—advocate escalation to biologic agents (e.g., tumor necrosis factor (TNF) inhibitors (TNFi) or interleukin-17 (IL-17) inhibitors) in patients exhibiting persistent disease activity or inadequate responses to NSAID therapy.^8^^,^^9^

Despite the widespread use of TNFi in axSpA, approximately 30%-50% of patients either fail to achieve a satisfactory clinical response—due to primary or secondary inefficacy—or discontinue therapy over time owing to loss of efficacy or adverse events (AEs).^10^^,^^11^ Following TNFi failure or in biologic-naïve patients, IL-17 inhibitors are generally preferred. However, under the reimbursement policies of the Turkish Social Security Institution (SGK), patients who have received only IL-17 inhibitor therapy are not eligible to switch directly to upadacitinib. Consequently, most patients become eligible for Janus kinase (JAK) inhibition only after prior exposure to TNFi and/or interleukin-17 inhibitor (IL-17i), resulting in a particularly treatment-refractory subgroup. This healthcare system context further underscores the critical need for real-world evidence (RWE) to guide clinical practice.

Janus kinase inhibitors represent an innovative oral treatment option in axSpA, characterized by fast therapeutic onset. These agents exert their effects by modulating the JAK–STAT signaling cascade, which transmits signals from major cytokines such as IL-6, IL-23, IFN-γ, and TNF-α that are central to disease pathogenesis.^12^^,^^13^ Upadacitinib is a selective JAK1 inhibitor, specifically designed to maximize anti-inflammatory efficacy while minimizing off-target effects by limiting inhibition of JAK2 and JAK3, thereby offering a potentially improved safety profile.^14^ The efficacy of upadacitinib in axSpA has been demonstrated through the SELECT-AXIS clinical development program. In the SELECT-AXIS 1 trial, patients with AS showed significant improvements in ASAS40 response rates, magnetic resonance imaging (MRI) inflammation scores, and functional outcomes.^15^ Similarly, the SELECT-AXIS 2 study confirmed comparable benefits in patients with nr-axSpA, including those previously treated with TNFi.^16^ Data from long-term extension trials indicate that upadacitinib sustains its therapeutic efficacy over a 2-year period, without significant changes in its established safety profile.^17^ However, randomized controlled trials (RCTs), by virtue of their highly controlled settings and selective patient populations, may not fully capture the heterogeneity and comorbidity burden observed in real-world patients with axSpA.

Real-world evidence is particularly valuable in this context, as it allows evaluation of treatment performance in complex, refractory populations often excluded from RCTs. Registry-based studies such as RHADAR^18^ and recent comparative analyses (e.g., Baraliakos et al., 2025)^19^ provide important benchmarks for interpreting outcomes of JAK inhibitors, including upadacitinib, in routine practice. While the effectiveness of JAK inhibitors on peripheral arthritis has been well documented through RWE over the past years, current knowledge regarding their impact on axial inflammation primarily stems from phase 3 clinical trials. Thus, real-world data from Türkiye—where TNFi failure is a prerequisite for JAK inhibitor access—are essential to address this critical gap.

Moreover, patients who experience failure with TNFi often exhibit a higher disease burden, reduced physical function, and diminished response rates to subsequent biologic therapies. In this challenging subgroup, the value of RWE becomes even more critical for informing treatment decisions and optimizing clinical outcomes.^20^ To address this gap in the literature, a 52-week real-world study was conducted in a tertiary care center in Türkiye to evaluate the effectiveness and safety of upadacitinib in patients with axSpA who had experienced prior failure with at least TNFi. Primary objectives included the longitudinal assessment of changes in disease activity indices, functional scores, and patient-reported outcomes. Secondary objectives focused on evaluating treatment persistence and the safety profile, including AEs.

## Methods

### Study Design and Setting

This was a retrospective, single-center, real-world observational study conducted in Denizli State Hospital a tertiary referral center for inflammatory rheumatic diseases in western Türkiye. The study period extended from October 27, 2023, to November 15, 2024. The study protocol was approved by the local Non-Interventional Clinical Research Ethics Committee of Pamukkale University (Approval No: E-60116787-020-615511; Date: November 27, 2024), and the study was conducted in accordance with the principles of the Declaration of Helsinki.The requirement for informed consent was waived by the local ethics committee due to the retrospective design of the study.

### Study Population

Eligible participants were adults (≥18 years) who fulfilled the ASAS classification criteria for axSpA. Inclusion criteria were a confirmed diagnosis of axSpA for at least six months; initiation of upadacitinib therapy due to inadequate response or intolerance to at least one TNFi; and availability of follow-up data for up to 52 weeks. Patients with coexisting peripheral arthritis were not excluded to reflect the frequent axial-peripheral overlap observed in real-world axSpA.

Exclusion criteria included: Active or chronic infections; New York Heart Association class III-IV heart failure; demyelinating diseases; history of malignancy within the last 5 years; and incomplete medical records.

All participants underwent comprehensive baseline screening, including tuberculin skin test or interferon-gamma release assay, hepatitis B and C virus and human immunodeficiency virus serologies, and pregnancy testing where applicable. One patient who conceived during treatment was closely monitored, and pregnancy outcome data were collected for safety documentation.

### Treatment Protocol and Data Collection

All patients received upadacitinib 15 mg orally once daily. Dose modifications were not permitted; however, temporary treatment interruptions due to AEs were allowed at the discretion of the treating physician. Clinical and laboratory parameters were assessed retrospectively at baseline and at weeks 12, 24, and 52.

Baseline variables included demographics, smoking and alcohol status, body mass index (BMI), human leukocyte antigen B27 (HLA-B27) status, presence of MRI-confirmed sacroiliitis, axSpA subtype (radiographic or non-radiographic), prior biologic exposure (TNFi, IL-17i), and comorbidities.

### Outcome Measures

Effectiveness outcomes were assessed using validated instruments:

- Bath Ankylosing Spondylitis Disease Activity Index (BASDAI)
- Ankylosing Spondylitis Disease Activity Score (ASDAS)
- Bath Ankylosing Spondylitis Functional Index (BASFI)
- Bath Ankylosing Spondylitis Metrology Index (BASMI)
- Visual Analog Scale for pain (VAS-pain)
- ASAS20, ASAS40, and ASAS70 response criteria

Low disease activity (LDA) was defined as BASDAI <4 and/or ASDAS <2.1, and very LDA as BASDAI <2 and/or ASDAS <1.3.

Safety outcomes included the incidence and type of AEs and serious AEs, reasons for treatment discontinuation, and pregnancy outcomes. Adverse events were additionally reported as incidence rates per 100 patient-years (PY) to allow comparison with other studies.

### Handling of Missing Data

For continuous variables, the Last Observation Carried Forward method was applied. For binary outcomes (e.g., ASAS responses), the non-responder imputation (NRI) method was used. These approaches help mitigate attrition bias, but no formal sensitivity analyses were performed, which remains a methodological limitation.

### Statistical Analysis

Descriptive statistics were used to summarize baseline characteristics. Time-dependent changes in continuous variables were analyzed using paired Student’s *t*-tests or Wilcoxon signed-rank tests, depending on normality assessed by the Kolmogorov–Smirnov test. To adjust for multiple testing across secondary endpoints, the Benjamini–Hochberg false discovery rate (BH–FDR) correction was applied, and *q*-values were reported in tables.

Kaplan–Meier survival analysis was performed to estimate treatment persistence, and the number at risk was reported at each timepoint. Cox proportional hazards models were constructed to assess predictors of treatment discontinuation, with covariates including age, BMI, HLA-B27 status, number of prior TNFi, and prior IL-17i exposure. Logistic regression analyses were additionally performed to explore baseline predictors of achieving LDA at week 24.

All statistical analyses were performed using SPSS version 22.0 (IBM SPSS Corp.; Armonk, NY, USA). A 2-sided *P*-value <.05 was considered statistically significant.

### Sample Size Estimation

Sample size estimation was based on the SELECT-AXIS 1 trial, which demonstrated a 27% absolute difference in ASAS40 responses between upadacitinib and placebo at week 14. Accordingly, a minimum of 42 patients was required to achieve 80% power at a 2-sided *α* = 0.05. Although this reference trial included biologic-naïve patients, the effect size from that study was adopted in the absence of published RWE-based power calculations for biologic-experienced populations. This constitutes a limitation of the design, but it was considered the most pragmatic approach. To enhance statistical power and reflect real-world practice, 45 patients were included in the final analysis.

## Results

### General Findings

A total of 45 patients with axSpA who had failed at least one TNFi were included in the analysis. All patients fulfilled the ASAS classification criteria and initiated upadacitinib therapy in routine clinical practice. Baseline characteristics are summarized in Table 1. The mean age was 48.1 ± 9.0 years, 53.3% were male, and 68.9% were HLA-B27 positive. The MRI-confirmed sacroiliitis was present in 95.6% of the cohort. Coexisting peripheral arthritis was observed in 31.1% of patients, while extra-musculoskeletal manifestations included psoriasis (11.1%), uveitis (8.9%), and inflammatory bowel disease (8.9%). Prior biologic exposure consisted of 1 TNFi in 55.6%, 2 TNFi in 28.9%, and ≥3 TNFi in 15.6% of patients, while 28.9% had previously received an IL-17 inhibitor. The mean baseline BASDAI was 6.50 ± 0.93, BASFI 5.84 ± 0.49, BASMI 3.63 ± 0.33, Health Assessment Questionnaire (HAQ) 0.83 ± 0.41, and VAS-pain 7.21 ± 1.04, reflecting high disease activity.

### Longitudinal Changes in Disease Activity and Function

**Figure 1.**
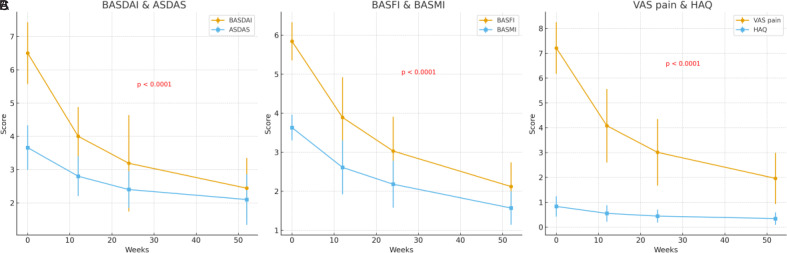
Changes in clinical parameters over 52 weeks of upadacitinib treatment. (A) BASDAI and ASDAS scores declined significantly at weeks 12, 24, and 52 (*P* < .0001). (B) BASFI and BASMI scores improved markedly, indicating better functional capacity and spinal mobility. (C) Patient-reported outcomes including VAS pain and HAQ scores demonstrated progressive improvement across all time points. Error bars represent standard deviations. Statistical significance is based on paired *t*-tests or Wilcoxon signed-rank tests depending on data distribution.

Significant improvements across all primary and secondary outcomes were observed over the 52-week follow-up (Table 2, Figure 1). The BASDAI decreased from 6.50 ± 0.93 at baseline to 2.44 ± 0.91 at week 52 (*P* < .0001, *q* < 0.0001). The ASDAS declined from 3.66 ± 0.67 to 2.10 ± 0.76 (p < 0.001). The BASFI improved from 5.84 ± 0.49 to 2.12 ± 0.62, and BASMI from 3.63 ± 0.33 to 1.57 ± 0.43 (both *P* < .0001, *q* < 0.0001).

Patient-reported outcomes also showed marked benefit: mean VAS-pain decreased from 7.21 ± 1.04 to 1.96 ± 1.03, and HAQ improved from 0.83 ± 0.41 to 0.34 ± 0.25 (both *P* < .0001, *q* < 0.0001). Joint counts demonstrated parallel improvement, with tender joint count reduced from 3.44 ± 3.43 to 0.53 ± 1.33 and swollen joint count from 1.00 ± 1.43 to 0.06 ± 0.34 at week 52 (*P* < .0001 each). All *P* values are reported in Table 2, and multiple testing was adjusted using Benjamini–Hochberg correction.

### Assessment of SpondyloArthritis International Society Response Rates

**Figure 2.**
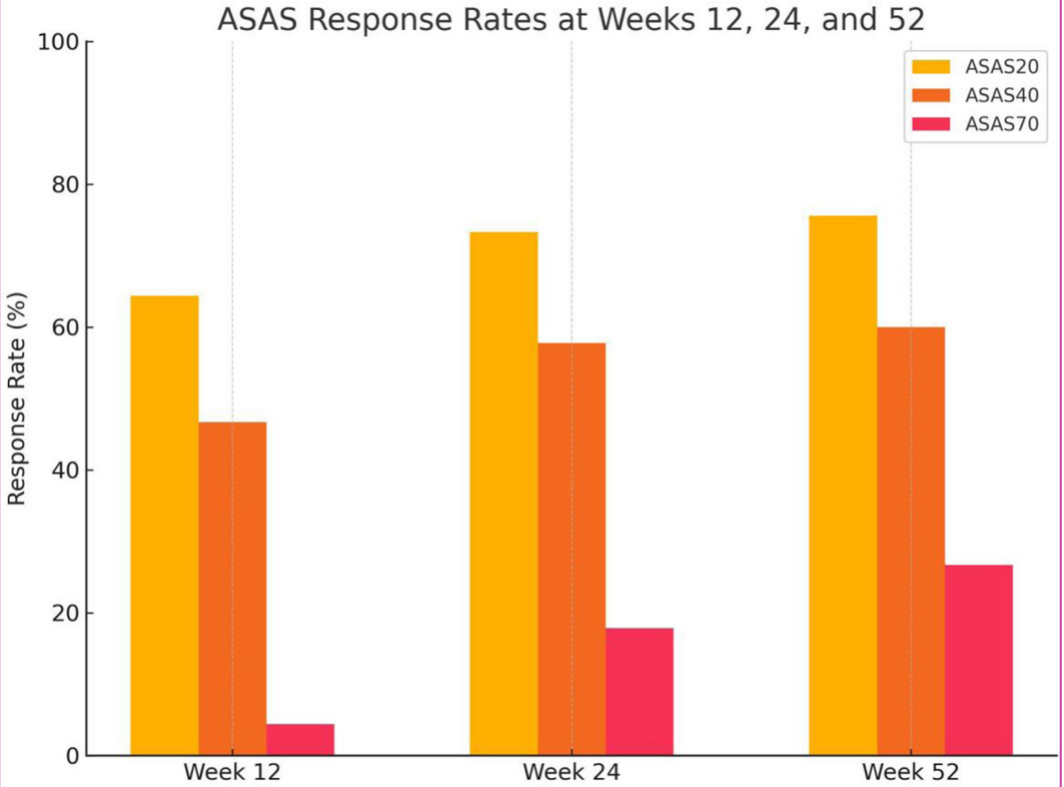
ASAS response rates at Weeks 12, 24, and 52. Proportion of patients achieving ASAS20, ASAS40, and ASAS70 response criteria at weeks 12, 24, and 52 of upadacitinib treatment in a real-world cohort of axial spondyloarthritis (axSpA) patients (n = 45). ASAS20 responses were observed in 64.4%, 73.3%, and 75.6% of patients at weeks 12, 24, and 52, respectively. Corresponding ASAS40 response rates were 46.7%, 57.8%, and 60.0%, while ASAS70 responses were achieved by 4.4%, 17.8%, and 26.7% of patients. Response rates were calculated using non-responder imputation. The figure illustrates the early and sustained clinical effectiveness of selective JAK1 inhibition in a TNFi-experienced axSpA population under routine care conditions.

Assessment of SpondyloArthritis International Society responses steadily increased and were sustained through week 52 (Table 3, Figure 2). At week 12, ASAS20/40/70 responses were achieved by 64.4% (29/45), 46.7% (21/45), and 4.4% (2/45) of patients, respectively. At week 24, response rates increased to 73.3% (33/45), 57.8% (26/45), and 17.8% (8/45). By week 52, ASAS20, ASAS40, and ASAS70 responses were attained in 75.6% (34/45), 60.0% (27/45), and 26.7% (12/45) of patients, respectively.

### Treatment Retention and Discontinuation

**Figure 3.**
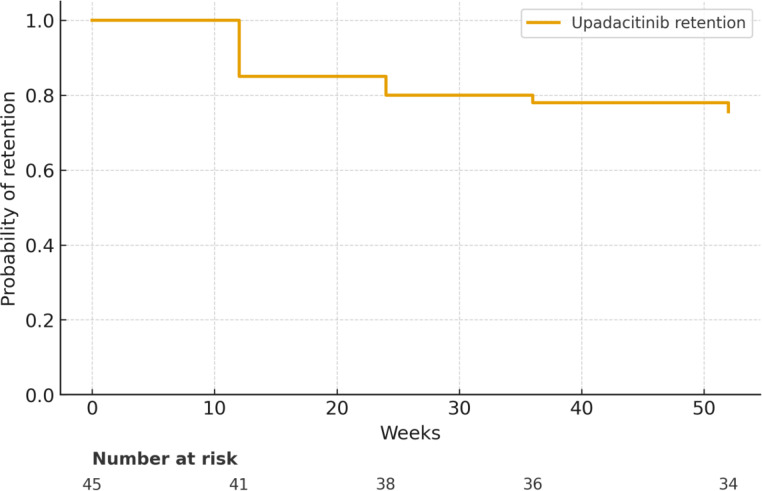
Kaplan–Meier survival curve illustrating the probability of treatment retention with upadacitinib over a 52-week period in patients with axial spondyloarthritis (axSpA). The cumulative retention rate was estimated using non-parametric survival analysis. At week 52, 34/45 patients (75.6%) remained on treatment. Censored observations (i.e., patients continuing treatment without discontinuation) were included. Numbers at risk are shown below the x-axis. This analysis highlights the long-term tolerability and persistence of selective JAK1 inhibition in routine clinical practice.

At week 52**, **75.6% of patients (34/45) remained on upadacitinib therapy, comprising 29 who continued without any AE (64.4%) and 5 who experienced an AE but remained on treatment (11.1%). Kaplan–Meier analysis (Figure 3) estimated a cumulative 52-week drug retention rate of 71.1%, with the number at risk displayed at each timepoint.

Overall, 11 patients (24.4%) discontinued treatment: 2 due to AEs (4.4%), 5 due to treatment failure (11.1%; 3 primary, 2 secondary), 3 due to patient preference (6.7%), and 1 due to pregnancy (2.2%) (Table 4). Adverse events leading to discontinuation included elevated liver enzymes (n = 2).

Cox proportional hazards analysis (Supplementary Table 2) identified prior TNFi exposure (HR 3.02, 95% CI 1.31–6.95, *P* = .0093) and prior IL-17 inhibitor therapy (HR 3.88, 95% CI 1.04-14.49, *P* = .0434) as significant predictors of earlier discontinuation.

### Responder Versus Non-Responder Analyses

Baseline comparisons between responders and non-responders at week 52 (Supplementary Table 1) showed that higher BMI was significantly associated with non-response for both ASAS20 (*P* = .0060) and ASAS40 (*P* = .0349). Prior IL-17 inhibitor exposure was more frequent among non-responders, although this association did not reach statistical significance in responder analyses. Other baseline characteristics—including age, sex, HLA-B27 status, peripheral arthritis, and extra-musculoskeletal manifestations—were comparable between groups. In contrast, Cox regression analysis (Supplementary Table 2) identified prior IL-17 inhibitor use (HR 3.88; 95% CI 1.04-14.49) and a history of multiple TNFi therapies (HR 3.02; 95% CI 1.31-6.95) as independent predictors of earlier treatment discontinuation, suggesting a differential impact on treatment persistence vs. clinical response. These findings highlight obesity and prior biologic exposure as potential determinants of treatment outcomes.

### Safety Profile

During a total exposure of 38.34 PY, no serious infections, venous thromboembolism, or malignancies were observed. The most common AEs were transient elevations in liver enzymes (n = 2; 5.22 per 100 PY) and increased creatine kinase (n = 2; 5.22 per 100 PY). Additional events included cytopenia (n = 1), acneiform eruptions (n = 1), and esophagitis (n = 1), each occurring at 2.61 per 100 PY (Supplementary Table 3). Adverse events are presented as incidence per 100 PY to facilitate comparability with other studies. One patient discontinued therapy due to pregnancy, which resulted in an uncomplicated live birth.

## Discussion

This 52-week real-world study conducted in a Turkish axSpA cohort provides pragmatic evidence on the effectiveness and safety of upadacitinib in patients with prior TNFi failure. While the retrospective, single-center design and modest sample size warrant cautious interpretation, the study addresses a critical knowledge gap by evaluating a highly refractory subgroup in a healthcare system where reimbursement policies strictly require documented TNFi failure before JAK inhibitor initiation. This policy context, characterized by universal prior TNFi exposure and additional IL-17 inhibitor exposure in a subset of patients, defines a treatment-resistant population with significant unmet clinical needs.

Consistent with phase 3 SELECT-AXIS trials, upadacitinib induced rapid and sustained improvements across multiple disease domains, including BASDAI, ASDAS, BASFI, BASMI, pain, and HAQ.^15^^,16^ Reductions in BASDAI (−4.15) and ASDAS (−1.56) over 52 weeks are particularly notable given the prior TNFi exposure and comorbidity burden in our cohort. Functional gains, reflected by approximately 60% and 49% reductions in BASFI and BASMI, underscore the potential of JAK1 inhibition to improve physical capacity and spinal mobility in daily practice.^21^^,^^22^

The ASAS responses increased progressively, with ASAS40 achieved in 60.0% and ASAS70 in 26.7% at week 52. These trajectories mirror and, in some respects, exceed outcomes from SELECT-AXIS 1 and 2,^15^^,^^16^ while being broadly aligned with contemporary RWE. For instance, registry-based analyses from RHADAR and a recent comparative study by Baraliakos et al (2025) reported meaningful responses to upadacitinib after TNFi switch, supporting our findings. Importantly, while early ASAS70 rates were modest, prolonged treatment enabled deeper responses in a subset of patients, emphasizing the value of persistence on therapy.^18^^,^^19^

Treatment retention in the study was 71.1% at 1 year, closely paralleling SELECT-AXIS 2 extension data^16^ and in line with RHADAR registry findings, where 2-year survival rates for JAK inhibitors were approximately 62.8% compared to 79.6% for TNFi and 72.6% for IL-17i.^18^ Although adherence was relatively high (~70%), the 11.1% rate of treatment failure and 28.9% overall discontinuation highlight the challenges of managing refractory disease in real-world settings. Cox regression analysis identified prior biologic exposure, particularly multiple TNFi or prior IL-17i use, as predictors of shorter drug survival, consistent with prior reports of attenuated responses in biologic-experienced patients.^20^^,^^23^

Responder vs. non-responder analyses revealed that higher BMI was significantly associated with lower ASAS responses (*P* < .01), a finding that resonates with prior evidence linking obesity to reduced biologic efficacy in axSpA.^24^ Smoking, sex, and HLA-B27 status were not significantly associated with treatment outcomes in the dataset, though larger cohorts are required to confirm these trends.

The safety profile was consistent with previous clinical trial and extension data.^12^^,^^17^^,^^25^ No malignancies, venous thromboembolic events, or serious infections were observed. Reported AEs were predominantly mild and manageable (elevated liver enzymes, CK increases, acneiform eruptions). Adverse events were additionally expressed as incidence per 100 PY to enable comparison with registry data, yielding rates similar to those reported in long-term studies.^19^ Importantly, 1 patient discontinued therapy due to pregnancy, which resulted in an uncomplicated live birth. Given the paucity of data on JAK inhibitor safety in pregnancy, this case contributes incremental but clinically relevant information.

Several limitations must be acknowledged. First, the retrospective, single-center design increases susceptibility to selection and reporting bias. Second, imaging-based endpoints (MRI inflammation, structural progression) were not assessed. Third, the small sample size (n = 45) restricts statistical power and may amplify chance findings. Fourth, the lack of a comparator arm precludes evaluation of relative efficacy vs. TNFi or IL-17i. Finally, although NRI and multiple testing correction were applied, no sensitivity analyses were conducted, which remains a methodological limitation.

In summary, this real-world study provides clinically relevant evidence that upadacitinib can achieve sustained disease control, functional improvement, and acceptable safety in TNFi-refractory axSpA patients in Türkiye. By situating these results within the unique reimbursement environment and by contextualizing them alongside both pivotal RCTs and RWE, the study strengthens the external validity of JAK1 inhibition in routine practice. Future prospective, multicenter studies are warranted to confirm these findings, explore long-term cardiovascular and thromboembolic safety, and further define predictors of response in difficult-to-treat populations.^26^

This 52-week real-world study provides evidence that upadacitinib offers clinically meaningful and durable improvements in disease activity, function, and patient-reported outcomes in Turkish patients with axSpA who had failed prior TNFi therapy. Treatment persistence was favorable, and the safety profile remained consistent with pivotal trials, with no unexpected AEs identified. While the retrospective design, single-center setting, modest sample size, and absence of imaging outcomes limit generalizability, the results highlight the therapeutic value of JAK1 inhibition in a uniquely treatment-refractory cohort shaped by national reimbursement policies. Future multicenter, prospective studies with larger populations and extended follow-up are warranted to confirm these findings, further define predictors of response, and evaluate long-term cardiovascular and thromboembolic safety.

## Figures and Tables

**Table 1.** Baseline Characteristics of the Cohort (N = 45)

Parameter	Value
N (patients)	45
Age, years (mean ± SD)	48.1 ± 9.0
BMI, kg/m^2^ (mean ± SD)	28.3 ± 3.7
HLA-B27 positivity, n (%)	31 (68.9)
Peripheral arthritis, n (%)	14 (31.1)
Psoriasis, n (%)	5 (11.1)
Uveitis, n (%)	4 (8.9)
Inflammatory bowel disease, n (%)	4 (8.9)
Prior TNFi = 1, n (%)	25 (55.6)
Prior TNFi = 2, n (%)	13 (28.9)
Prior TNFi ≥ 3, n (%)	7 (15.6)
Prior IL-17 inhibitor exposure, n (%)	13 (28.9)
BASDAI at baseline, mean ± SD	6.50 ± 0.93
BASFI at baseline, mean ± SD	5.84 ± 0.49
BASMI at baseline, mean ± SD	3.63 ± 0.33
HAQ at baseline, mean ± SD	0.83 ± 0.41
VAS_pain at baseline, mean ± SD	7.21 ± 1.04
TJC at baseline, mean ± SD	3.44 ± 3.43
SJC at baseline, mean ± SD	1.00 ± 1.43

Data are presented as mean ± standard deviation (SD) or n (%), unless otherwise specified.

BASDAI, Bath Ankylosing Spondylitis Disease Activity Index; BASFI, Bath Ankylosing Spondylitis Functional Index; BASMI, Bath Ankylosing Spondylitis Metrology Index; HAQ, Health Assessment Questionnaire; HLA B27, human leukocyte antigen B27; IBD, inflammatory bowel disease; IL 17, interleukin 17; TJC, tender joint count; TNFi, tumor necrosis factor inhibitor; SJC, swollen joint count.

**Table 2.** Longitudinal Changes from Baseline at Weeks 12, 24, and 52 (Paired Analysis with BH–FDR)

Outcome	Week	N Pairs	Baseline (Mean ± SD)	Follow-up (Mean ± SD)	Change (Mean ± SD)	*P*	*q* (BH-FDR)	Test
BASDAI	12	45	6.50 ± 0.93	4.00 ± 0.88	−2.50 ± 1.04	<.0001	2.343369016843959e-19	Paired *t*-test
BASDAI	24	39	6.54 ± 0.86	3.19 ± 1.45	−3.35 ± 1.47	<.0001	1.597895018719089e-07	Wilcoxon
BASDAI	52	33	6.59 ± 0.82	2.44 ± 0.91	−4.15 ± 0.94	<.0001	1.138575888959641e-21	Paired *t*-test
BASFI	12	43	5.84 ± 0.49	3.89 ± 1.03	−1.95 ± 1.13	<.0001	7.29583259003575e-14	Paired *t*-test
BASFI	24	39	5.81 ± 0.47	3.03 ± 0.88	−2.78 ± 0.97	<.0001	2.343369016843959e-19	Paired *t*-test
BASFI	52	34	5.84 ± 0.47	2.12 ± 0.62	−3.72 ± 0.82	<.0001	1.684686882144441e-22	Paired *t*-test
BASMI	12	43	3.63 ± 0.33	2.61 ± 0.69	−1.01 ± 0.77	<.0001	5.968558980384842e-13	Wilcoxon
BASMI	24	39	3.61 ± 0.34	2.18 ± 0.60	−1.44 ± 0.72	<.0001	7.639755494892598e-12	Wilcoxon
BASMI	52	33	3.64 ± 0.34	1.57 ± 0.43	−2.07 ± 0.61	<.0001	9.430701572093617e-19	Paired *t*-test
HAQ	12	45	0.83 ± 0.41	0.55 ± 0.33	−0.28 ± 0.23	<.0001	2.075267661085437e-10	Paired *t*-test
HAQ	24	39	0.91 ± 0.38	0.44 ± 0.26	−0.47 ± 0.32	<.0001	6.600624286952744e-11	Paired *t*-test
HAQ	52	34	0.94 ± 0.39	0.34 ± 0.25	−0.60 ± 0.30	<.0001	8.351951809486205e-13	Paired *t*-test
SJC	12	45	1.00 ± 1.43	0.38 ± 0.98	−0.62 ± 1.09	.0006	0.0005532345345920751	Wilcoxon
SJC	24	39	1.05 ± 1.41	0.03 ± 0.16	−1.03 ± 1.40	.0001	0.000115266747614813	Wilcoxon
SJC	52	34	1.21 ± 1.45	0.06 ± 0.34	−1.15 ± 1.37	.0001	0.000115266747614813	Wilcoxon
TJC	12	45	3.44 ± 3.43	1.49 ± 1.82	−1.96 ± 2.28	<.0001	4.239591587015725e-06	Wilcoxon
TJC	24	39	3.87 ± 3.47	1.15 ± 2.06	−2.72 ± 2.68	<.0001	4.239591587015725e-06	Wilcoxon
TJC	52	34	3.76 ± 3.36	0.53 ± 1.33	−3.24 ± 2.87	<.0001	6.144111429305547e-06	Wilcoxon
VAS_pain	12	45	7.21 ± 1.04	4.08 ± 1.48	−3.13 ± 1.70	<.0001	2.236693598899567e-15	Paired *t*-test
VAS_pain	24	38	7.36 ± 1.05	3.01 ± 1.34	−4.34 ± 1.60	<.0001	2.193882537453602e-07	Wilcoxon
VAS_pain	52	34	7.40 ± 1.08	1.96 ± 1.03	−5.43 ± 1.26	<.0001	2.037268131971359e-10	Wilcoxon

Paired analyses compare baseline with the indicated week using normality-checked tests (paired *t*-test if normality not rejected on within-patient change; otherwise Wilcoxon signed-rank). Multiple testing was controlled using the Benjamini–Hochberg false discovery rate (reported as *q*-values). Only patients with both baseline and the respective follow-up value were included in the paired analysis (complete-case per timepoint). ASDAS was intentionally excluded per the analysis plan.

BASDAI, Bath Ankylosing Spondylitis Disease Activity Index; BASFI, Bath Ankylosing Spondylitis Functional Index; BASMI, Bath Ankylosing Spondylitis Metrology Index; BH–FDR, Benjamini–Hochberg false discovery rate; HAQ, Health Assessment Questionnaire; SJC, swollen joint count; TJC, tender joint count; VAS, visual analogue scale.

**Table 3.** ASAS Response Rates at Weeks 12, 24, and 52

Time Point	ASAS20 (n, %)	ASAS40 (n, %)	ASAS70 (n, %)
Week 12	29/45 (64.4)	21/45 (46.7)	2/45 (4.4)
Week 24	33/45 (73.3)	26/45 (57.8)	8/45 (17.8)
Week 52	34/45 (75.6)	27/45 (60.0)	12/ 5 (26.7)

ASAS20/40/70, Assessment of SpondyloArthritis International Society criteria for 20%, 40%, and 70% improvement.

**Table 4.** Reasons for Treatment Discontinuation by Week 52

Reason for Discontinuation	Number of Patients (n)	Percentage (%)
Continued without adverse event	29	64.4
Adverse event occurred but continued	5	11.1
Primary treatment failure	3	6.7
Secondary treatment failure	2	4.4
Patient decision	3	6.7
Discontinuation due to AE	2	4.4
Pregnancy	1	2.2

Distribution of reasons for treatment discontinuation during the 52-week follow-up (n = 45). Percentages are calculated as a proportion of the full cohort. Patients who remained on upadacitinib at week 52 are separated into those with and without any recorded adverse event during follow-up.

**Supplementary Table 1.** Baseline characteristics by responder status at Week 52 across ASAS20/40/70 (NRI)

Variable	Responders	Non-responders	P-value
1a) ASAS20 (Week 52, NRI)			
Age, years (mean ± SD)	49.5 ± 8.7	44.2 ± 9.1	0.1465
BMI, kg/m^2^ (mean ± SD)	29.1 ± 3.8	25.9 ± 2.2	0.0060
HLA-B27: Positive	22 (66.7%)	9 (75.0%)	0.7254
Prior TNFi count: 1	19 (57.6%)	6 (50.0%)	0.4574
Prior TNFi count: 2	8 (24.2%)	5 (41.7%)	0.5884
Prior TNFi count: ≥3	6 (18.2%)	1 (8.3%)	0,6112
Prior IL-17 exposure: Exposed	7 (21.2%)	6 (50.0%)	0.0757
Peripheral arthritis: Yes	9 (27.3%)	5 (41.7%)	0.4703
Psoriasis: Yes	4 (12.1%)	1 (8.3%)	1.0000
Uveitis: Yes	3 (9.1%)	1 (8.3%)	1.0000
IBD: Yes	3 (9.1%)	1 (8.3%)	1.0000
**1b) ASAS40 (Week 52, NRI)**			
Age, years (mean ± SD)	49.5 ± 8.9	45.1 ± 8.9	0.2150
BMI, kg/m^2^ (mean ± SD)	29.1 ± 3.9	26.5 ± 2.7	0.0349
HLA-B27: Positive	20 (64.5%)	11 (78.6%)	0.4921
Prior TNFi count: 1	17 (54.8%)	8 (57.1%)	0.5322
Prior TNFi count: 2	8 (25.8%)	5 (35.7%)	0.5642
Prior TNFi count: ≥3	6 (19.4%)	1 (7.1%)	0.7354
Prior IL-17 exposure: Exposed	6 (19.4%)	7 (50.0%)	0.0724
Peripheral arthritis: Yes	8 (25.8%)	6 (42.9%)	0.3074
Psoriasis: Yes	4 (12.9%)	1 (7.1%)	1.0000
Psoriasis: No	27 (87.1%)	13 (92.9%)	
Uveitis: Yes	3 (9.7%)	1 (7.1%)	1.0000
IBD: Yes	3 (9.7%)	1 (7.1%)	1.0000
**1c) ASAS70 (Week 52, NRI)**			
Age, years (mean ± SD)	49.8 ± 9.2	47.4 ± 9.0	0.5144
BMI, kg/m^2^ (mean ± SD)	29.0 ± 3.2	28.0 ± 3.9	0.2759
HLA-B27: Positive	9 (69.2%)	22 (68.8%)	1.0000
Prior TNFi count: 1	9 (69.2%)	16 (50.0%)	0.4128
Prior TNFi count: 2	2 (15.4%)	11 (34.4%)	0.4411
Prior TNFi count: ≥3	2 (15.4%)	5 (15.6%)	0.6533
Prior IL-17 exposure: Exposed	3 (23.1%)	10 (31.2%)	0.7253
Peripheral arthritis: Yes	5 (38.5%)	9 (28.1%)	0.5024
Psoriasis: Yes	3 (23.1%)	2 (6.2%)	0.1359
Uveitis: Yes	2 (15.4%)	2 (6.2%)	0.5672
IBD: Yes	1 (7.7%)	3 (9.4%)	1.0000

Notes: Non-responder imputation (NRI) applied to classify Week-52 ASAS outcomes; missing values counted as non-response. Continuous variables were compared using Mann–Whitney U; categorical variables using Fisher’s exact test or χ² as appropriate. Percentages are calculated within responder and non-responder groups for each endpoint.

**Supplementary Table 2:** . Cox proportional hazards for treatment discontinuation (drug survival)

Covariate	HR (95% CI)	P-value
Age	0.95 (0.87–1.03)	0.2368
BMI	0.94 (0.78–1.12)	0.4602
HLA_B27_positivity	1.82 (0.43–7.80)	0.4185
Prior_TNFi_count	3.02 (1.31–6.95)	0.0093
IL17_exposed	3.88 (1.04–14.49)	0.0434

Notes: Cox proportional hazards model; reference for smoking is ‘Never’. HR > 1 indicates higher hazard of discontinuation.

**Supplementary Table 3:** . Adverse events (excluding pregnancy): incidence per 100 patient-years

Preferred term	Events (n)	Incidence per 100 PY	95% CI (per 100 PY)
Any Adverse Event (Excl. Pregnancy)	7	18.26	7.34–37.62
Ck↑	2	5.22	0.63–18.84
Cytopenia	1	2.61	0.07–14.53
Acne	1	2.61	0.07–14.53
Esophagitis	1	2.61	0.07–14.53
Liver Enzyme Elevation	2	5.22	0.63–18.84

Notes: Pregnancy is not coded in the AE field and is excluded by definition. Total exposure = 38.34 PY. Exact Poisson 95% CIs shown.
